# Polymorphisms of two loci at the oxytocin receptor gene in populations of Africa, Asia and South Europe

**DOI:** 10.1186/s12863-015-0323-8

**Published:** 2016-01-06

**Authors:** Polina R. Butovskaya, Oleg E. Lazebny, Evgeniya M. Sukhodolskaya, Vasily A. Vasiliev, Daria A. Dronova, Juliya N. Fedenok, Aracelli Rosa, Elena N. Peletskaya, Alexey P. Ryskov, Marina L. Butovskaya

**Affiliations:** Vavilov Institute of General Genetics, Russian Academy of Sciences, Moscow, Russia; Koltzov Institute of Developmental Biology, Russian Academy of Sciences, Moscow, Russia; Institute of Gene Biology, Russian Academy of Sciences, Moscow, Russia; Institute of Ethnology and Anthropology, Russian Academy of Sciences, Moscow, Russia; Unitat d’Antropologia, Departament de Biologia Animal, Facultat de Biologia, Universitat de Barcelona (UB), Barcelona; Centro de Investigaciones Biomédicas en Red de Salud Mental (CIBERSAM), Madrid, Spain; Institut de Biomedicina de la Universitat de Barcelona (IBUB), Barcelona, Spain; Genelex, Inc, 3101, Suite 100, Western Avenue, Seattle, WA 98121 USA; Russian State University for the Humanities, Moscow, Russia

**Keywords:** Oxytocin receptor gene, SNP, rs53576, rs2254298, Parental behavior, Hadza, Datoga, Ob Urgic, Catalans

## Abstract

**Background:**

The oxytocin (OT) system is known to be implicated in the regulation of complex social behavior, particularly empathy and parenting. The goal of this study was to estimate the gender and population differences in polymorphisms of two oxytocin receptor gene SNPs, rs53576 and rs2254298, in four populations.

**Results:**

These data were compared with each other and with 14 samples from the corresponding regions retrieved from the 1000 Genomes database. Low level of heterozygosity was observed for both SNPs in all populations in this study (rs53576: Catalonian, Hobs = 0.413; Hadza, Hobs = 0.556; sr2254698: Khanty-Mansi, Hobs = 0.250; Datoga, Hobs = 0.550). The amount of variance due to regional variability was almost equal for both SNPs (rs53576: F_RT_ = 0.086, rs2554298: F_RT_ = 0.072), whereas variance for the population level of variability was twice bigger for rs2554298 (rs53576: F_ST_ = 0.127, rs2554298: F_ST_ = 0.162). Pairwise coefficients of fixation demonstrate that the Hadza were well differentiated from other African populations except of Datoga, the Datoga were weakly differentiated from other African origin populations, the Ob Ugric people were extremely differentiated from all other populations. Catalans were extremely differentiated of Asian populations.

**Conclusions:**

It is hypothesized on the base of spatial distribution of the evolutionary novel A alleles of the both OXTR gene loci, that the spread of alleles of rs22542298 and rs53376 SNPs may be associated to some extant with manipulation of parental investment in humans.

**Electronic supplementary material:**

The online version of this article (doi:10.1186/s12863-015-0323-8) contains supplementary material, which is available to authorized users.

## Background

The oxytocin (OT) system is known to be implicated in the regulation of complex social behavior, such as empathy, affiliated behavior and parenting, and response to social stress [1]. OT has been related to attachments between parents and children, intimate partners, relatives and friends, and its level is reported to be stable over time within individuals [2]. Several studies have indicated an association of OT with mental disorders that are characterized by impaired social behavior, such as autism, anxiety, and depression [3]. OT is a peptide of nine amino acids that is produced in the hypothalamus and released into both the brain and bloodstream. Functioning as both a neurotransmitter and hormone, oxytocin’s targets are widespread and include the hypothalamus, amygdala, hippocampus, brainstem, heart, uterus, and regions of the spinal cord that regulate the autonomic nervous system, especially the parasympathetic branch [1, 4, 5].

OT is known to regulate social bonds in animals [6], and CD38−/− knockout mice had both decreased plasma OT level and significant social impairments, including poorer maternal nurturing and less effective social behaviors [7]. Recent studies suggest that oxytocin may have similar functions in human well-being [8]. It was found that oxytocin increases trust, generosity [9] and empathy accuracy [10]. Recent neuroimaging evidence suggests that those brain areas involved in emotion processing and social danger monitoring, especially in the amygdala, were found to have decreased activation [11, 12]. Given the known heritability of empathy in humans [13], these data further suggest that genetic variation in the oxytocinergic signaling may have a role in empathy modulation [14]. Variation in the oxytocin receptor (OXTR) gene may partly explain individual differences in OT-related social behavior [15]. In humans, the oxytocin receptor is encoded by the OXTR gene which has been localized to human chromosome 3p25 and has four exons and three introns [5]. Two single nucleotide polymorphisms (SNPs) in the third intron have been suggested to be particularly promising candidates to explain differences in oxytocinergic functioning: rs53576 (G–A) and rs2254298 (G–A) [16].

According to the data for the rs53576 polymorphism of the *OXTR* gene in 12 nations, the nations with higher frequency of *A* allele (Japan, China, Korea) are more collectivistic in comparison with those having higher frequency of *G* allele (USA, UK, Australia, Canada, Netherlands, Italy, Sweden, Germany, Finland) but at the same time the last ones seem to be more predisposed to major depressive disorder [17]. Although, these data should be accepted with cautious. Same authors demonstrated recently that *AA* and *GG* carriers of *OXTR* rs53576 demonstrated different brain activity, using functional magnetic resonance imaging (MRI), in reaction to racial ingroup and outgroup faces that received painful or non-painful stimulations, and suggested that these differences are linked to implicit attitude and altruistic motivation [18].

The highly representative study of 7723 British mother’s behavior failed to show the relationship between rs53576 *OXTR* genotype and emotional loneliness [19]. While in other studies, the *G* allele carriers in comparison with homozygotes *AA* appeared to be more prosocial [20]. Positive association of the *OXTR* gene with autism has been demonstrated in the Chinese Han population [21].

There is less evidence for the SNP rs2254298 associations with behavior qualities and personality features, although Feldman and colleagues have shown that this polymorphism may be associated with plasma levels of oxytocin, particularly individuals with *GG* having lower levels of OT [22]. Other authors have associated depression and anxiety with the OXTR rs2254298 polymorphism in adults [23], and with autism in children [24]. While the *A* allele of *OXTR* rs2254298 was associated with attachment security in the non-Caucasian infants [25].

OXTR demonstrate contrasting patterns of expression in brains in monogamous and polygamous species of voles, particularly, the OXTR in the septum may be associated with social behavior, whereas those in the BNST/amygdala may be associated with parental behavior [26]. Supposedly, in species with high density of OXTR in the nucleus accumbens both females and males demonstrate parental care [27]. But whether any genetic basis exist for above mentioned differences (e.g., differential sex-specific parental care) between populations, practicing monogamous and polygamous mating patterns, still remained to be tested.

It is challenging to look for such associations in human ethnics practicing monogamous and polygamous reproduction behavior. We know two African tribes that fit this criterion and one Eurasian nation that can serve as a control. The Hadza are hunter-gatherers from Northern Tanzania. Population size is about 1500 individuals. They are relatively egalitarian and monogamous and have nominal leadership [28–30]. Hadza men compete in the form of successful hunting, and female mate choice is important [31, 32]. Male reproductive output is positively associated with hunting skills and informal leadership [33, 34]. The Datoga are traditional seminomadic pastoralists of Tanzania [35]. Population size is about 100,000 individuals. They are polygynous, and horizontally divided into generation sets with clear wealth stratification [36]. The social status and number of wives and children sired by a man are correlated with his wealth [37]. To address violence within families or clans, the Datoga have developed judicial institutions based on customary laws [36, 38] that include public assembly, clan moots, and women’s and neighborhood councils. Using a system of fines and ostracizing of habitual aggressors, the Datoga manage within-tribal violence [39].

Ob-Ugric people (Khanty and Mansi) settled on the territory of Russia in Western Siberia and occupied the basins of the Ob and the Irtysh rivers, including their tributaries. According to the census conducted in 2010, Ob-Ugric peoples are just over 43 thousand people (Khanty – 31,000 and Mansi – 12,000). Their languages belong to Ugric subgroup of Finno-Ugric group of the Uralic language family. Many of them are still practicing traditional occupations, such as fishing, hunting reindeer herding, and gathering [40]. Despite the fact that currently most of the Ob-Ugric people living in villages, they still practice nomadic reindeer herding. They are patrilineal, marriages are patrilocal and basically monogamous [41].

The aim of our study was to investigate population specificity in the distributions of allelic frequencies of two SNPs in *OXTR* gene (rs53576, rs2254298). To meet this goal we selected three populations from traditional culture represented different regions of the world: East Africa (Hadza, Datoga), Asia (Ugric people from Western Siberia), and one modern population of South Europe (Catalonian). Populations we selected for the purpose of our study are of special interest, because they may drop some light on the functionality of rs53576 and rs2254298 polymorphisms. Two of them, Hadza and Ugric people are mainly monogamous, while Datoga are polygynous. Spanish sample has been used for comparative purposes as one of the modern industrial population, monogamous.

## Results

Allele and genotype frequency distributions obtained for our four samples were compared with each other as well as to 14 samples (live in and originated from Africa: Yoruba in Ibadan, Nigeria; Luhya in Webuye, Kenya; African Ancestry in Southwest US (AASUSA); Latin America: Colombian in Medellin; Puerto Rican in Puerto Rico; Mexican Ancestry in Los Angeles, California (MexAn); Asia: Han Chinese in Bejing (Han1), China; Southern Han Chinese (Han2), China; Japanese in Tokyo, Japan; live in and originated from Europe: Finnish in Finland; British in England and Scotland; Iberian populations in Spain; Utah residents with Northern and Western European ancestry (UNWE)) from the corresponding region retrieved from the 1000 Genomes database. The allele and genotype distributions did not differ significantly between men and women in all samples with p-values ranged from 0.04 (rs53576 and rs2254298) in Spanish to 0.75 (rs2254298) and 0.79 (rs53576) in Datoga (Additional file 1).

Data on distribution of allelic and genotype frequencies of the two studied SNP loci are presented in Table 1. Genotype frequencies for rs53576 and rs2254298 in the all 18 samples including four studied were in accordance with the Hardy–Weinberg equilibrium with Benjamini and Hochberg correction (Table 2), corrected p-value was higher 0.05 in both cases (uncorrected p-values for rs53576 and rs2254298 varied from 0.030 to 0.948 and from 0.073 to 0.963, correspondingly.

**Table 1:** Allelic and genotype frequencies of SNPs rs53576 and rs2254298 of oxytocin receptor gene in world populations

		Frequencies					
		Allele			Genotype		
rs53576							
Population	Number	A	G	Number	AA	AG	GG
African All*	1194	0.224	0.776	597	0.057	0.333	0.610
Americans of African Ancestry in SW USA	122	0.287	0.713	61	0.082	0.410	0.508
Luhya in Webuye, Kenya	198	0.211	0.789	99	0.052	0.320	0.629
Yoruba in Ibadan, Nigeria	216	0.193	0.807	108	0.045	0.295	0.659
**Hadza**, **Tanzania**	270	0.426	0.574	135	0.148	0.556	0.296
**Datoga**, **Tanzania**	388	0.302	0.698	194	0.077	0.448	0.474
American All*	524	0.343	0.657	262	0.116	0.453	0.431
Colombians from Medellin, Colombia	188	0.333	0.667	94	0.083	0.500	0.417
Mexican Ancestry from Los Angeles USA	128	0.386	0.614	64	0.136	0.500	0.364
Puerto Ricans from Puerto Rico	208	0.300	0.700	104	0.127	0.345	0.527
Asian All*	1144	0.684	0.316	662	0.472	0.423	0.105
Han Chinese in Bejing, China	206	0.716	0.284	103	0.505	0.423	0.072
Southern Han Chinese	210	0.670	0.330	105	0.480	0.380	0.140
**Khanty and Mansi**	700	0.571	0.429	350	0.349	0.446	0.206
Japanese in Tokyo, Japan	208	0.663	0.337	104	0.427	0.472	0.101
European All*	1990	0.361	0.639	995	0.132	0.459	0.409
Utah Residents (CEPH) with Northern and Western European Ancestry	198	0.294	0.706	99	0.082	0.424	0.494
Finnish in Finland	198	0.446	0.554	99	0.151	0.591	0.258
British in England and Scotland	182	0.393	0.607	91	0.191	0.404	0.404
Iberian Population in Spain	214	0.250	0.750	107	0.143	0.214	0.643
**Catalonian**	984	0.307	0.693	492	0.091	0.431	0.478
Toscani in Italia	214	0.327	0.673	107	0.102	0.449	0.449
**rs2254298**							
African All*	1112	0.238	0.762	561	0.053	0.370	0.577
Americans of African Ancestry in SW USA	122	0.287	0.713	61	0.049	0.475	0.475
Luhya in Webuye, Kenya	198	0.191	0.809	99	0.041	0.299	0.660
Yoruba in Ibadan, Nigeria	216	0.256	0.744	108	0.068	0.375	0.557
**Hadza**, **Tanzania**	226	0.274	0.726	113	0.053	0.442	0.504
**Datoga**, **Tanzania**	360	0.303	0.697	180	0.078	0.450	0.472
American All*	524	0.240	0.760	262	0.083	0.315	0.602
Colombians from Medellin, Colombia	188	0.225	0.775	94	0.083	0.283	0.633
Mexican Ancestry from Los Angeles USA	128	0.250	0.750	64	0.121	0.258	0.621
Puerto Ricans from Puerto Rico	208	0.245	0.755	104	0.036	0.418	0.545
Asian All*	968	0.322	0.678	484	0.101	0.441	0.458
Han Chinese in Bejing, China	206	0.320	0.680	103	0.103	0.433	0.464
Southern Han Chinese	210	0.355	0.645	105	0.140	0.430	0.430
Japanese in Tokyo, Japan	208	0.287	0.713	104	0.056	0.461	0.483
**Khanty and Mansi**	344	0.154	0.846	172	0.029	0.250	0.721
European All*	2318	0.107	0.893	1159	0.016	0.182	0.802
Utah Residents (CEPH) with Northern and Western European Ancestry	198	0.076	0.924	99	0.000	0.153	0.847
Finnish in Finland	198	0.081	0.919	99	0.000	0.161	0.839
British in England and Scotland	182	0.090	0.910	91	0.011	0.157	0.831
Iberian Population in Spain	214	0.107	0.893	107	0.000	0.214	0.786
**Catalonian**	1312	0.168	0.832	656	0.037	0.262	0.701
Toscani in Italia	214	0.173	0.827	107	0.051	0.245	0.704

*Total data for world region (1000 Genomes database http://www.1000genomes.org/) are calculated including information on our samples presented in bold letters

**Table 2:** The Hardy–Weinberg equilibrium for 18 populations, including our data on Hadza, Datoga, Ob Ugric people and Catalonian

Pop	DF	ChiSq	Prob	ChiSq	Prob
		**rs53576**		**rs2254298**	
**Hadza**	1	2.499	0.114	1.401	0.237
**Datoga**	1	0.810	0.368	0.780	0.377
AASUSA	1	0.121	0.728	0.901	0.342
Lugya, Kenya	1	0.004	0.948	1.507	0.220
Yoruba, Nigeria	1	0.036	0.850	0.273	0.601
Colombia	1	2.714	0.099	0.550	0.458
MexAn	1	0.040	0.841	3.218	0.073
Puerto_Rico	1	1.614	0.204	2.137	0.144
**Khanty**-**Mansi**	1	2.835	0.092	0.288	0.592
Han1	1	0.086	0.769	0.002	0.963
Han2	1	2.509	0.113	0.792	0.373
Japanese	1	1.137	0.286	1.035	0.309
**Catalans**	1	0.081	0.776	2.415	0.120
UNWE	1	0.010	0.920	0.665	0.415
Finnish	1	4.714	0.030	0.873	0.350
British	1	0.168	0.682	0.150	0.698
Iberian	1	0.974	0.324	2.629	0.105
Toscanian	1	1.251	0.263	2.282	0.131

our data presented in bold letters; probabilities were corrected in accordance with Benjamini-Hochberg: q > 0.05

We tested the close physical location of the two SNPs – 2143 bp between them with the linkage disequilibrium test with the option of unknown phases. The results obtained were in accordance with physical linkage of the two loci in three out of four studied populations. The probabilities of free allelic combination between the two locus studied were as follows: the Hadza: 0.0000003, the Ob Ugric people: 0.0005; the Catalans: 0.000006. However, in the case of Datoga, these two loci demonstrated free recombination: 0.48. Suggestively this may be due to the bottleneck effect which took place in the 19th century when Datoga were kicked out from Ngoro-Ngoro region by Maasai, the other possibility is related to the hot spot of recombination located between the SNPs. Finally, this may be due to our sample, although it relatively large (178 individuals). This fact remained to be investigated in the future as currently we are unable to explain this result.

For the SNP rs53576 the frequency of *A* allele in Hadza was much higher compared to other African samples, presented in Tables 2 and 4, and Datoga significantly differed only from Yoruba. Accordingly, the *AA* genotype frequency was the highest in Hadza. The frequency of *A* allele in Ob Ugric people was significantly lower compared to other Asian samples presented, same is true for the *AA* genotype frequency (Tables 1 and 3). Also, Ob Ugric people significantly differed from all Europeans populations presented in this study, but in this case the frequencies of *A* allele as well as *AA* genotype were higher in Ugric people compared to Europeans. The Catalonian sample for *A* allele and *AA* genotype frequencies fell within the variation of other European groups, with the exception of Finnish population (G = 17.74, d.f. = 1, *p* = 0.00003), as well as demonstrated no differences from Columbians, Puerto Ricans and Utah residents of European origin (Tables 1 and 3), besides, they did not differ from Datoga (Tables 1 and 3). For the SNP rs2254298 of the *OXTR* gene, the *A* allele and *AA* genotype frequencies for Hadza sample were comparable with the other African groups (Tables 1 and 3), this was true for Datoga as well with the exception of differences between Datoga and Luhya from Kenya (G = 4.26, d.f. = 1, *p* = 0.039). On the contrary, the *A* allele and *AA* genotype frequencies were significantly lower in the Ob Ugric sample compared to other Asian groups: Han1, G = 21.55, d.f. = 1, *p* = 0.000004; Han2, G = 34.63, d.f. = 1, *p* = 0.000000004; Japanese, G = 12.25, d.f. = 1, *p* = 0.0005 (Tables 1 and 3). Catalonian sample have similar frequencies of the *A* allele and *AA* genotype with Iberic and Toscani samples, as well as with Colombians and Puerto Ricans, but different compared to Finnish (G = 9.92, d.f. = 1, *p* = 0.0016), British (G = 8.70, d.f. = 1, *p* = 0.0032) and Utah samples (G = 12.90, d.f. = 1, *p* = 0.0003) (Tables 1 and 3).

**Table 3:** Results of G-test for comparison of allele and genotype frequencies in the populations studied

		rs53576			rs2254298		
Pop1	Pop2	G	df	Chi p	G	df	Chi p
Hadza	Datoga	10.731	1	0.001	0.546	1	0.460
Hadza	AASUSA	8.890	1	0.003	0.000	1	1.000
Datoga	AASUSA	0.436	1	0.509	0.462	1	0.497
Hadza	Luhya	20.577	1	0.000	1.536	1	0.215
Datoga	Luhya	3.692	1	0.055	4.255	1	0.039
Hadza	Yoruba	33.179	1	0.000	0.849	1	0.357
Datoga	Yoruba	10.126	1	0.001	3.035	1	0.082
Khanty-Mansi	Han1	10.234	1	0.001	21.548	1	0.000
Khanty-Mansi	Han2	4.982	1	0.026	34.627	1	0.000
Khanty-Mansi	Japanese	4.566	1	0.033	12.254	1	0.000
Spanish	Colombian	0.000	1	1.000	1.320	1	0.251
Spanish	MexAn	6.610	1	0.010	5.960	1	0.015
Spanish	Puerto_Rican	0.444	1	0.505	1.838	1	0.175
Spanish	UNWE	0.000	1	1.000	12.903	1	0.000
Spanish	Finnish	17.743	1	0.000	9.919	1	0.002
Spanish	British	3.602	1	0.058	8.695	1	0.003
Spanish	Iberian	0.000	1	1.000	1.453	1	0.228
Spanish	Toscanian	0.000	1	1.000	0.000	1	1.000

Legend: Pop1 and Pop2 are populations to be compared for their frequency distributions, G – G-criterion, d.f. – degrees of freedom, Chi p – probability value obtained on the bases of χ^2^ distribution

Low level of heterozygosity was observed for the both SNPs in all populations in this study. European populations are characterized with the lowest values of observed heterozygosity (Table 1).

**Fig. 1:**
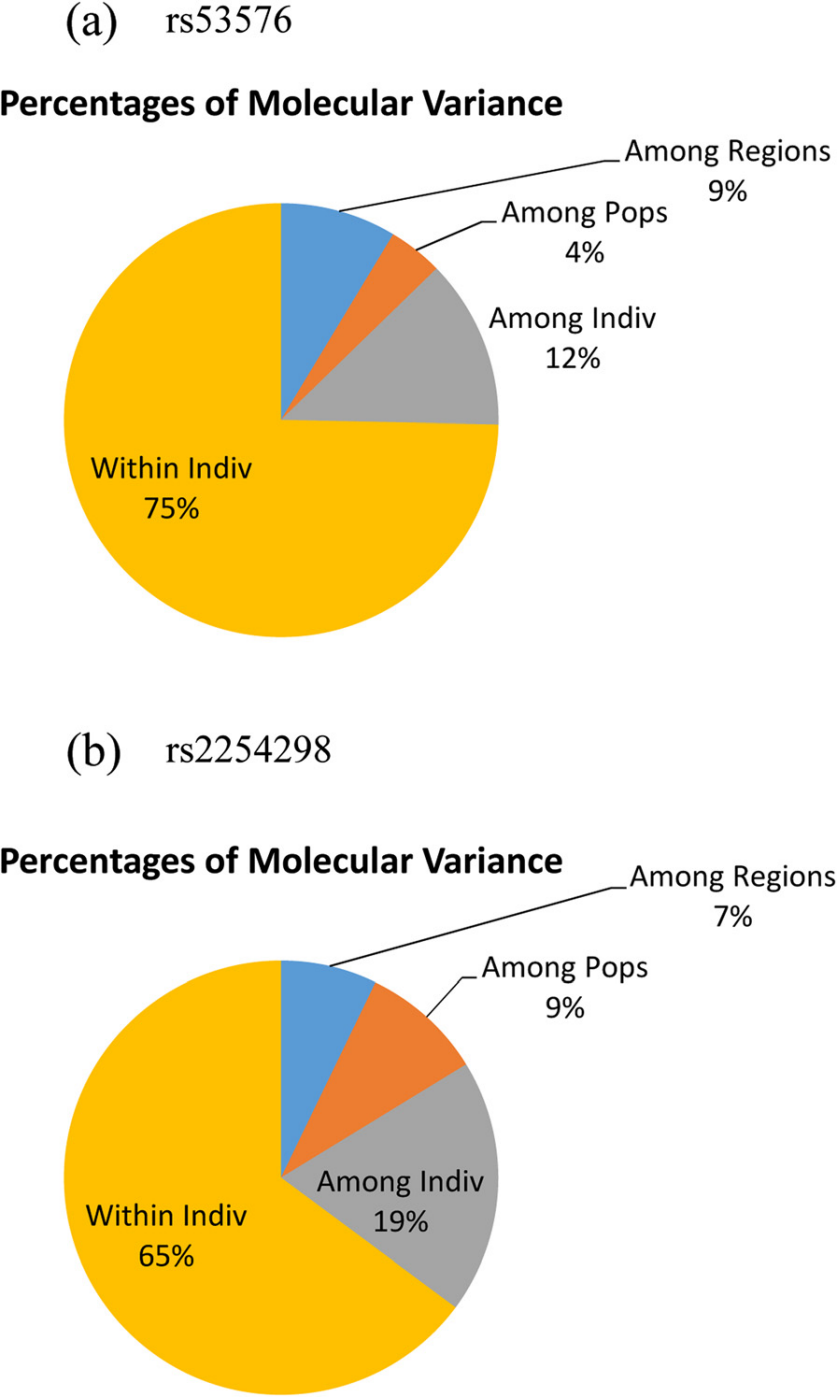
Diagrams of percentages of molecular variance for rs53576 (**a**) and rs2254298 (**b**)

Percentages of molecular variance for both SNPs are presented on Fig. 1. The amount of variance due to regional variability is almost equal for both SNPs (rs53576: F_RT_ = 0.086, *p* = 0.001; rs2554298: F_RT_ = 0.072, *p* = 0.001), whereas variance for the population level of variability was twice bigger for rs2554298 (rs53576: F_ST_ = 0.127, *p* = 0.001; rs2554298: F_ST_ = 0.162, *p* = 0.001). Coefficient of fixation demonstrates the degree of population differentiation (Table 4). According to these values, the Hadza are well differentiated from other African populations except of Datoga, and also from the rest of populations except of Mexicans (from LA). The Datoga are weakly differentiated if at all from other African origin populations, including Afro-Americans from our samples, a bit stronger differentiation is observed for Datoga vs Iberians and Toscanians. The Ob Ugric people are extremely differentiated from all other populations (from 0.093 with the Hadza to 0.266 with the Yoruba). Catalonians are moderately differentiated from others except of Asian populations (Han from Bejing, 0.17; Han from South China, 0.17, Japanese from Tokyo, 0.14) (Table 4).

**Table 4:** Pairwise F_ST_ values for our studied samples with samples from the 1000 genome database

	Datoga	AASUSA	Luhya	Yoruba	Colombian	MexAn	Puerto_Rican	Khanty-Mansi	Han1	Han2	Japanese	Catalonian	UNWE	Finnish	British	Iberian	Toscanian
Hadza	0.017	0.039	0.065	0.083	0.045	0.016	0.049	0.093	0.083	0.068	0.066	0.088	0.094	0.072	0.071	0.065	0.053
Datoga		0.004	0.019	0.026	0.017	0.019	0.017	0.169	0.144	0.122	0.121	0.073	0.070	0.088	0.067	0.040	0.029
Khanty-Mansi		0.217	0.247	0.266	0.221	0.174	0.227		0.169	0.164	0.166	0.244	0.258	0.213	0.228	0.236	0.227
Catalonian		0.063	0.068	0.083	0.053	0.058	0.055	0.244	0.170	0.165	0.140		0.062	0.068	0.056	0.053	0.051

## Discussion

**Fig. 2:**
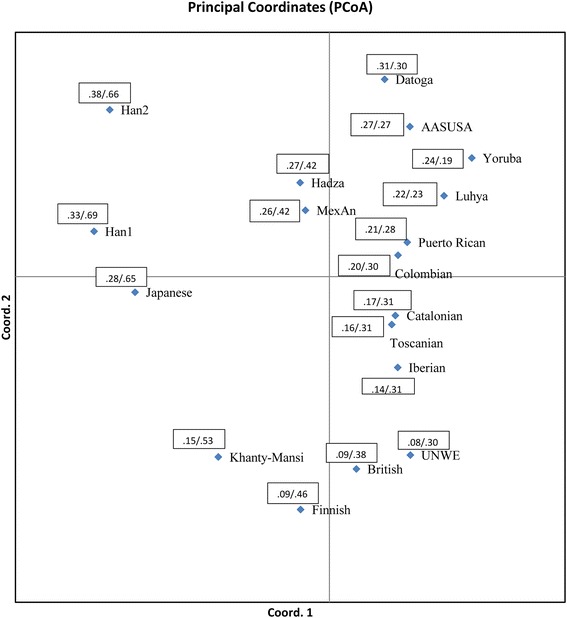
Ordination of the 18 samples by means of PCA. Two numbers located near markers stand for the *A* allele frequencies of the rs2254298 (*before slash*) and the rs53376 (*after slash*)

The frequency of *A* allele of rs2254298 in the *OXTR* gene differs a lot between the populations presented in this paper. In samples of Europeans, particularly of northern origin, the frequency of this allele is minimal, while in Asian populations the *A* allele frequency is much higher. Africans demonstrating intermediate frequencies but closer to Asian ones. In regards to the rs53376, Europeans and Africans demonstrated comparable *A* allele frequencies, and in Asian populations it’s frequencies were much higher. The principal component analysis conducted on the basis of allele frequencies of both SNPs generally reveal the distribution of 18 populations in accordance with their geographic distributions (Fig. 2). Principal Coordinate 1 reflected the geographic distribution from West to East, with Yoruba to the most West and Han from Beijing to the East. Coordinate 2 reflects the North to South distribution with Finnish being the Northern, following by British and Utah Residents (CEPH) with Northern and Western European Ancestry. Ob Ugric people being close to Finnish. The Datoga and African Americans and South Chinese located on the most South (Fig. 2). Right top quarter accumulated all African samples, as well as Colombians, and Puerto Ricans. Right low quarter united European populations with the only exception of Finnish sample. All Asian populations located in the Eastern half of space between coordinates 1 and 2 (Fig. 2). The position of Hadza sample in the space of 1 and 2 coordinated deserves special attention. Hadza outstand from other African groups, locating in direction to Asian populations. One of the possible explanations is that current combination of 2 SNP frequencies of *OXTR* gene may be due to bottleneck effect. It was found recently by Tishkoff with co-authors [42], that the proportion of polymorphic sites as quantified by q, indicated that formally the effective size of Hadza populations could be estimated as about 9200–20,900 individuals, meantime, currently their population has been substantially decreased in size (currently being about 1500 individuals).

A tendency can be easily seeing on the Fig. 2 concerning frequencies of the *A* alleles of both loci of the *OXTR* gene. More simple tendency is related to the rs2254298, which consists in decreasing of frequency of *A* allele when moving down along the Principal Ordinate 2 (from South to North). As for spatial distribution of the populations in regards to the rs53376, African populations are characterized by similar frequencies of *A* allele, except of Hadza. In Europeans, frequencies of *A* allele at the rs53376 were at least twice higher compared to the rs2254298. The highest frequencies of the *A* allele of rs53376 were found in Asians.

The obtained spatial distribution of allele frequencies of both loci of the *OXTR* gene may be explained in accordance with general information on associations between the level of plasma oxytocin and parental touch and warmness. As demonstrated for the rs2254298 SNP, the *A* allele being associated with lower plasma oxytocin and lower related parental attachment [22]. According to previous studies, this allele is specific for humans, as primates don’t have it, while *G* allele is the ancestral for this SNP [43]. Another *OXTR* gene SNP rs53376 has been associated with individual differences in maternal and empathic behavior as well, with *A* allele identified as a risk one [44]. Given the fact of distribution of these alleles world-wide in modern humans, we suggest that it is either beneficial, or at least neutral for it’s carriers. It is hypothesized, that selection in favor of *A* alleles of rs2254298 and rs53376 SNPs may be associated to some extant with manipulation of parental investment in humans [45–47], favoritisms towards sons, being one of possible examples. Practice of infanticide in many human populations being another example of such parental manipulation strategies [48]. Obvious preferences for sons result in sex-biased infanticide, with girls being main targets, are known to be practiced in China, Republic of Korea, India and other countries of East and South Asia [49]. Such behavior may be developed in the process of human evolution along with extension of offspring dependence and development of childhood and adolescence phases of life cycle [50]. Asian samples presented in our study had the lowest frequencies of GG genotypes on rs53576. We suggest that possible increase in frequencies of *A* allele in this SNP was to some extant associated with intensive practice of selective infanticide for centuries in these populations. Of course we do not mean that infanticidal practice was the only factor favoring the distribution of *A* alleles. Different marriage patterns, associated with different parental investment, different intensity of same sex cooperation in everyday life may be enumerated as other possible factors associated with population differences in distribution of *A* and *G* alleles of rs22542298 and rs53376 SNPs of *OXTR* gene in humans. In our sample those populations, known to practice selective infanticide had lowest frequencies of rs53376 *G* allele (China, Japan), while those with highest frequencies of this allele were those, with highest mothers investment in child care (Datoga, Nigerians, Luhya from Kenya, Columbians) and same sex male cooperation. As demonstrated by other authors, on within - population level *GG* rs53376 SNP male carriers in Han population of Singapore revealed significant association between *GG* genotype and low 2D:4D ratio (proxy to prenatal androgenization) with higher cognitive abilities in reading others emotional states [51].

## Conclusions

In this study, a geographic spatial distribution of the alleles of rs22542298 and rs53376 SNPs of the *OXTR* gene in human populations was revealed. This regularity in the allele distributions assumes effects of some factors on polymorphism of the *OXTR* gene. So long as the product of this gene is involved in the regulation of complex social behavior, we hypothesize that the spread of alleles of rs22542298 and rs53376 SNPs may be associated to some extant with manipulation of parental investment in humans.

## Methods

### Ethics approval and consent

Institutional approvals, including university (Moscow State University Ethics Committee for data collection in Russia, Ob-Ugric people, Hadza and Datoga of Tanzania), local governmental agencies (Tanzanian Commission for Science and Technology for data collection of Hadza and Datoga), were obtained prior to conducting this study. All subjects gave their informed, verbal consent prior to participation. Verbal consent was deemed appropriate given the low literacy rates among traditional Hadza and Datoga, and was specifically approved by university EC and Tanzanian agency. The study on Spanish population was approved by the Universitat Autónoma de Barcelona (Spain) Ethics Committee and confirmed to the Helsinki Declaration. All participants provided written informed consent.

### Participants

The present study included four samples of healthy individuals from different populations: 1) 353 Ob-Ugric people (Khanty and Mansi) from Khanty-Mansiysky Autonomous District of the Nothern-Western Siberia were collected in 2010 – 2011; 2) 135 adult Hadza and 196 adult Datoga were collected in 2006–2007 in the Lake Eyasi region of Northern Tanzania and 3) Catalonian sample, represented by 659 students have been collected at Barcelona University in 2010–2013 (Table 5).

**Table 5:** General information about samples

Population	Age (years)	Men	Women
Hadza	16–70	72	63
Datoga	16–70	104	92
Khanty-Mansi	16–71	127	226
Catalonian	17–51	157	502

### DNA analysis

All the participants provided Buccal samples. Genomic DNA was isolated using Diatom DNA Prep 200 (Isogen Lab, Moscow, Russia) (samples 1 and 2) and Real Extraction DNA kit (Durviz S.L.U., Valencia, Spain) (sample 3). DNA quality from all the samples was assessed by spectrophotometer readings (A260/280) using Nanodrop.

Two polymorphisms in the *OXTR* gene (rs53676 and rs2254298) were genotyped using Taqman 5′ exonuclease assay (Applied Biosystems). The probes for genotyping were ordered through the TaqMan SNP genotyping Assays (ID: C___3290335_10) and (ID: C__15981334_10) Applied Biosystems assay-on-demand service. The final volume was 5 μl, which contained 5 ng of genomic DNA, 2.5 μl of Taqman Master Mix and 0.25 μl of 40 genotyping assay. Polymerase chain reaction plates were read on an ABI PRISM 7900HT instrument and SDS v2.3 software (Applied Biosystems) was used for the genotype analysis of data.

### Statistical analyses

All population statistical data processing was carried out using GenAlEx software v6.5 (http://biology-assets.anu.edu.au/GenAlEx/Welcome.html): genotype and allele frequencies, tests for HWE, test of homogeneity, linkage disequilibrium test, estimations of heterozogosity and F_ST_ and their significances, AMOVA.

### Supporting data

The genotypic data for the all four studied populations are available by the following DOI: 10.6070/H4V40S6S. Data is deposited in LabArchives in a form that excludes any possibility of participant identification.

## Additional file

Additional file 1:
**Four studied populations.** (XLSX 62 kb)

## Abbreviations

CD38−/−: A strain of CD38-deficient mice
rs##: A reference SNP ID number is an identification tag assigned by NCBI to a group (or cluster) of SNPs that map to an identical location
BNST: Bed nucleus of the stria terminalis
HWE: Hardy-Weinberg equilibrium
AMOVA: Analysis of molecular variance
